# MicroRNA, an Antisense RNA, in Sensing Myeloid Malignancies

**DOI:** 10.3389/fonc.2017.00331

**Published:** 2018-01-30

**Authors:** Stephanie Rebecca Setijono, Hyog Young Kwon, Su Jung Song

**Affiliations:** ^1^Soonchunhyang Institute of Medi-bio Science, Soonchunhyang University, Cheonan-si, South Korea

**Keywords:** acute myeloid leukemia, microRNAs, microRNA-based therapeutics, myelodysplastic syndrome, myeloid malignancies

## Abstract

Myeloid malignancies, including myelodysplastic syndromes and acute myeloid leukemia, are clonal diseases arising in hematopoietic stem or progenitor cells. In recent years, microRNA (miRNA) expression profiling studies have revealed close associations of miRNAs with cytogenetic and molecular subtypes of myeloid malignancies, as well as outcome and prognosis of patients. However, the roles of miRNA deregulation in the pathogenesis of myeloid malignancies and how they cooperate with protein-coding gene variants in pathological mechanisms leading to the diseases have not yet been fully understood. In this review, we focus on recent insights into the role of miRNAs in the development and progression of myeloid malignant diseases and discuss the prospect that miRNAs may serve as a potential therapeutic target for leukemia.

## Introduction

### MicroRNAs (miRNAs) in Cancer

MicroRNAs are small non-coding RNAs with 19–22 nucleotides to control gene expression through binding to mRNA of their cognate target genes and thereby participate in numerous biological processes such as cell proliferation, differentiation, development, metabolism, apoptosis, survival, and hematopoiesis (1). *miRNA* is transcribed by RNA pol II/III to generate a primary miRNA followed by nuclear cleavage by the RNase III endonuclease Drosha and its binding to the double-stranded RNA-binding protein DGCR8 to form a precursor miRNA (pre-miRNA) (2, 3). Subsequently, the pre-miRNA is transported by Exportin-5/RanGTP to the cytoplasm to be further cleaved by the RNase III endonuclease Dicer, leaving an unstable miRNA duplex that unwinds. The 5′ guide strand containing the mature miRNA sequence is incorporated into a ribonucleotide silencing complex, while the 3′ passenger strand undergoes rapid degradation (4–6).

While miRNAs located within chromosomes deleted in cancer play roles as tumor suppressors, miRNAs located in genomic regions amplified in cancer function as oncogenes. Deregulated miRNAs found in both solid tumors and hematopoietic malignancies target the transcripts of essential protein-coding genes involved in tumorigenesis (7, 8). Fingerprints of miRNAs’ expression are linked to clinical and biological characteristics of tumors including tissue type, aggressiveness, and therapy response. Abnormal expression of pre-miRNA is also found in various types of human cancer. Because sequence abnormalities of *miRNAs’* genes and transcripts are also observed in the germline (8), the inherited subtle variations in miRNAs may have a great effect on the expression profiles of protein-coding genes in cancer.

### miRNAs in Hematological Malignancies

Hematological malignancies comprise a collection of heterogeneous diseases, all originating from cells of the bone marrow or lymphatic system. Hematological malignancies include leukemias, lymphomas, myelodysplastic syndromes (MDS), and myeloproliferative neoplasms (9). Myeloid malignancies are clonal disorders that are characterized by excessive proliferation, abnormal self-renewal, and/or differentiation blocks of hematopoietic stem cells (HSCs) and myeloid progenitor cells (10, 11). miRNA expression profiling in myeloid malignancies has revealed distinct signatures associated with diagnosis, stage classification, progression, prognosis, and response to treatment of leukemias (Table 1). miRNAs can be regulated by epigenetic modifiers including DNA methylation and histone modification in leukemias, suggesting that aberrant expression of miRNAs by epigenetic mechanisms may trigger hematopoietic cell transformation.

**Table 1:** MicroRNAs (miRNAs) in myeloid malignancies.

miRNA	Expression profiles in leukemias	Reference
miR-22	High in AML/high in MDS	(12, 13)
miR-99	High in AML	(14)
miR-128a	High in AML	(15)
miR-155	High in AML	(16)
miR-182	High in AML	(17)
miR-221/miR-222	High in AML	(18)
miR-4262	High in AML	(19)
miR-29	Low in AML	(20)
miR-34a	Low in AML	(21)
miR-34b	Low in AML	(22)
miR-137	Low in AML	(23)
miR-142-3p	Low in AML	(24)
miR-194-5p	Low in AML	(25)
miR-204	Low in AML	(18)
miR-217	Low in AML	(26)
miR-223	Low in AML with t(8;21)	(18, 27–29)
miR-302a	Low in AML	(30)
miR-451	Low in AML	(31)
miR-650	Low in AML	(32)
miR-125b	High in AML	(33, 34)
miR-192	Low in AML	(35, 36)
miR-193	Low in AML	(37)
miR-124	Low in AML	(38, 39)
miR-181a	Low in AML	(18, 40)
miR-196b	High in AML	(34, 41)
miR-21	High in acute lymphoblastic leukemia, high in MDS	(42, 43)
miR-17–92/miR-20	High in CML/high in MDS	(44, 45)
miR-10a	Low in CML, high in MDS	(18, 46)
miR-126	High in AML	(47, 48)
miR-155	High in MDS	(46)
miR-130	High in MDS	(46)
MiR-144/451	Low in MDS	(12, 49)
miR-146a	Low in MDS	(46)
miR-150	Low in MDS	(46)
let-7a	Low in MDS	(46)

*AML, acute myeloid leukemia; MDS, myelodysplastic syndromes*.

In this review, we focus on recent advances in understanding the roles of miRNA deregulation in the pathogenesis of myeloid malignancies and discuss the prospect that miRNAs may serve as potential therapeutic targets for leukemias.

## miRNA Deregulation in MDS

Myelodysplastic syndromes are HSC disorders characterized by ineffective hematopoiesis and a high risk of progression to acute myeloid leukemia (AML) (50). More than 70% of all human miRNAs are located within regions of recurrent copy-number alterations in MDS and AML cell lines (51). The targeted ablation of *Dicer1* in murine hematopoietic system leads to abnormal hematopoiesis and MDS, supporting the relevance of miRNA deregulation to the pathogenesis of MDS (52).

Several recent studies have addressed the role of miRNAs in MDS pathogenesis. Vasilatou et al. have shown that miR-17-5p and miR-20a, as members of the miR-17–92 cluster, repress the transcription factor E2F1, which is highly expressed in 67% of patients with MDS (44). Similarly, let-7a downregulates KRAS, which is aberrantly expressed in high-risk MDS (53, 54). A subset of miRNAs involved in stage-specific regulation of erythropoiesis are also deregulated in MDS (55). Overexpression of miR-181, miR-221, miR-376b, miR-125b, miR-155, or miR-130a inhibits erythroid cell growth (56), and this event might be responsible for disease-associated ineffective erythropoiesis. miR-155 targeting CEBPB and CSF1R is significantly upregulated in high-risk MDS (57). High expressions of miR-155, miR-126, and miR-130 in MDS restrain megakaryopoiesis and may account for higher frequency of thrombocytopenia observed during disease progression (46). However, recent evidence reveals that reduction of Rho family members by miR-155 contributes to impaired neutrophil migration in MDS (58). miR-21 expression has been found to be increased in MDS, and its interaction with SMAD7 mRNA leads to ineffective, MDS-like hematopoiesis *via* overactivating TGFβ signaling (42). In addition, serum miR-21 level appears to act as a potential non-invasive biomarker that predicts a response following treatment with hypo-methylating agents, such as azacytidine or decitabine in MDS patients (59). In contrast, decreased expression of the miR-144/451 members targeting the erythroid transcription factor GATA-1 is closely associated with high-risk MDS (12, 49). Overall, both the aberrant expression and the function of miRNAs are the important factors contributing to MDS pathogenesis and prognosis.

Despite significant amount of evidence demonstrating miRNA expression and role in tumorigenesis is available, a very few studies illustrate mechanisms of miRNA deregulation and related mechanisms underlying MDS. miR-22 has been found to be overexpressed in MDS patients with poorer survival outcome (60). Furthermore, transgenic mice expressing hematopoietic miR-22 exhibit decreased global 5-hydroxymethylcytosine levels and increased HSC self-renewal along with defective differentiation and develop MDS and myeloid leukemia over time. miR-22 directly targets the DNA demethylating enzyme ten-eleven-translocation 2 (TET2) and affects the epigenetic landscape in the hematopoietic compartment, while forced expression of TET2 suppresses the miR-22-induced malignant phenotypes. A significant inverse correlation between miR-22 and TET2 observed in MDS patients suggest the miR-22-TET2 regulatory network as a reliable factor for MDS pathogenesis (60, 61). A better understanding of miR-22 deregulation in MDS disease progression and AML transformation will provide insight into the mechanisms of MDS pathogenesis and provide new therapeutic strategies against leukemia transformation.

## Emerging Roles of miRNA Deregulation in the Pathogenesis of AML

Acute myeloid leukemia is characterized by the accumulation of immature myeloid cells in the bone marrow and shows genetic abnormalities including mutations and chromosomal translocations (10). Distinctive miRNA expression profiles have been demonstrated for cytogenetic subtypes and mutations in *CEBPA, FLT3*, and *NPM1* of AML (62–64). miRNA profiles are also associated with AML prognosis, underscoring the importance of miRNAs in AML (65). As such, miRNAs impact AML development and progression through targeting known oncogenes or tumor suppressors or collaborating with them to promote or suppress myeloid malignancy.

miR-9 has shown to be overexpressed in MLL-rearranged AML and play a critical oncogenic role in MLL fusion-mediated leukemogenesis (66). Ectopic expression of miR-9 blocks neutrophil development in myeloid cell lines and in murine primary lineage-negative bone marrow cells by inhibiting ETS-related gene (67). Also, miR-9 exerts its tumor-suppressive effects through the cooperation with let-7 to repress the oncogenic Lin28b/HMGA2 axis in AML (68).

miR-125b is upregulated in AML patients and blocks the differentiation of AML blast cells by directly targeting the cytoplasmic tyrosine-protein kinase FES that is expressed exclusively in myeloid cells (33). Overexpression of miR-125b also leads to a reduction in expression of the RNA binding protein Lin28A (69), which is known to play an important role in stem cell biology.

miR-181 and miR-128 target Lin28, leading to the progression of myeloid leukemia and differentiation blockage of hematopoietic cells to their lineage (15, 70, 71). The inhibition of miR-181 expression partially reverses the lack of myeloid differentiation in AML patients and in the mice implanted with CD34+ hematopoietic stem/progenitor cells (HSPCs) from AML patients (72).

The targeted miR-126 reduction in cell lines and primary AML samples results in decreased AML growth through inhibiting multiple components of the PI3K/AKT/mTOR pathway (47, 73, 74). The attenuated expression of miR-126 also leads to expand normal HSC (75), suggesting that miR-126 dictates opposing self-renewal outcomes in normal and leukemic HSC. Furthermore, both gain- and loss-of-function *in vivo* studies of miR-126 in murine models demonstrate that either overexpression or knockout of miR-126 promotes development of AML in mice (76). This result suggests that miR-126 plays a dual role in leukemogenesis and supports a new layer of miRNA regulation in AML.

Overexpressed miR-155 is associated with poor outcome in AML patients. miR-155 promotes FLT3-ITD-induced myeloproliferative disorder through inhibition of the interferon (IFN) response, inositol 5-phosphatase 1 (SHIP1), CEBPB, and PU.1, while it is upregulated in FLT3-ITD+ and MLL-rearranged AML (57, 77–80). These results suggest that miR-155 can collaborate with FLT3-ITD to promote myeloid cell expansion, and this involves a multi-target mechanism that includes repression of IFN signaling.

miR-22 is overexpressed in AML, and its aberrant expression correlates with silencing of TET2 in AML patients (60). Approximately 70% of miR-22 transgenic mice develop AML by 2 years of age. Also, miR-22 impairs the MLL-AF9-induced leukemogenesis through repressing CREB and Myc pathways and relieves the monocyte/macrophage differentiation and the growth of AML by targeting MECOM (81, 82). Therefore, in AML, miR-22 can be both oncogenic and tumor-suppressive, depending on the specific individual backgrounds (e.g., early HSCs versus the committed myeloid progenitors).

The accumulation of peroxiredoxin III caused by decreased miR-26a leads to a marked reduction in reactive oxygen species (ROS) in primary AML granulocyte samples (83). Growing evidence demonstrates that ROS plays a key role in regulating the balance between self-renewal and differentiation of HSCs (84). Thus, the reduced ROS levels might drive HSCs toward differentiation into myeloid lineage fates, providing a potential mechanism for miR-26a’s role as a tumor suppressor.

miR-29a appears to be significantly increased in peripheral blood mononuclear cells and bone marrow white blood cells from AML patients. Increased miR-29a promotes differentiation into granulocytes and monocytes, while reduction of miR-29a suppresses myeloid differentiation in leukemic cells (85). In myeloid leukemogenesis, c-Myc inhibits miR-29a expression, resulting in increased AKT2 and Cyclin D2 expressions in AML (86). Conversely, ectopic expression of miR-29a in murine HSPCs leads to acquisition of self-renewal capacity by myeloid progenitors, biased myeloid differentiation, and the development of a myeloproliferative disorder that progresses to AML (87).

miR-34a is downregulated in AML and induces apoptosis *via* inhibition of autophagy by targeting HMGB1 in leukemic cells (21). miR-34b plays a critical role in AML pathogenesis by targeting CREB, and its expression is repressed due to its promoter hypermethylation in AML patients (22). The methylation of the miR-124a family, including miR-124a-1 and miR-124a-3, is also observed in AML patients independently of their cytogenetic subtypes (88). It is also noted that epigenetic silencing of miR-124a is associated with the expression of EVI1 in AML (89, 90).

While studies of miR-125b suggested that it has oncogenic role in AML, miR-125a is considered as a tumor-suppressive miRNA. miR-125a expression in cytogenetically normal AML appear to be most decreased in favorable and intermediate prognostic populations and associated with decreased survival (91).

In the context of AML caused by toxic DNA interstrand crosslinks (ICLs), miR-139 and miR-199a have opposite roles in hematopoietic cell expansion and leukemogenesis (92). The levels of miR-139 and miR-199a are elevated with age in myeloid progenitors from the nucleotide excision repair gene (*Ercc1*)-deficient mice. Ectopic expression of miR-139 inhibits proliferation of myeloid progenitors, whereas increased miR-199a enhances proliferation of progenitors and augments the AML phenotype. This study supports the oncogenic role of miR-199a and also indicates that the elevated miR-139 as a tumor suppressor is involved in the defective hematopoietic function in ICL-induced AML.

miR-223 decreases cell proliferation and enhances cell apoptosis in AML *via* targeting FBW7 (93, 94). miR-223 was originally identified as a critical regulator in granulopoiesis and transactivated by NFI-A and C/EBPα in acute promyelocytic leukemia (27). AML1/ETO oncoprotein induces epigenetic silencing of miR-223 through directly binding to the *pre-miR-223* gene in AML (28). miR-223 targets E2F1 to inhibit cell cycle progression, thereby resulting in myeloid differentiation, and in turn, E2F1 represses miR-223 transcription, forming a negative feedback loop in AML (95–97). In summary, scientific evidence supporting the role of miRNAs in the pathogenesis of AML with proven tumor suppressors or oncogenic activities is becoming increasingly clear.

## miRNA-Based Therapeutics in Myeloid Malignancies

As the understanding of miRNA expression and action in myeloid malignancies continues to evolve, miRNAs have a great potential to serve as both the non-invasive biomarkers and a potential therapeutic target for leukemia. For example, miRNA expression signatures classify leukemias of uncertain lineage as either AML or acute lymphoblastic leukemia (98). miRNA expression profiles can also predict progression of MDS to AML (99) and survival outcome of AML patients (100, 101). Furthermore, circulating miRNAs have been recently demonstrated as an economical, non-invasive, and sensitive tool to monitor for minimal residual disease, which refers to the persistence of a small number of leukemic blasts in the bone marrow after chemotherapy and can ultimately cause disease relapse. Indeed, AML patients have a distinctive circulating miRNA expression profiles compared to healthy controls (102, 103), and an altered expression signature of serum miRNAs is observed after standard chemotherapy (104).

Yet, cancer therapy is based on a therapy targeting a single gene or pathway: “one target, one drug” model. A treatment effectively targeting multiple genes and pathways of cancer concomitantly may be an important innovation. Such an approach would not only more effectively suppress cancer cell growth but also would inhibit the common emergence of resistance in a single gene or pathway. miRNAs form a complex network where each miRNA can regulate multiple genes and pathways and each gene or pathway can be regulated by multiple miRNAs. Thus, miRNAs hold promising potential for “multi-targeted therapy” in cancer patients. To date, miRNA replacement therapy has largely made use of synthetic miRNA mimics to restore lost tumor suppressor expression (105). Restoration of lost tumor suppressor miRNAs using synthetic double-stranded RNAs (with a delivery agent) has been successful in preclinical models of leukemia. For example, the targeted delivery of miR-29b mimics by transferrin-conjugated lipid nanoparticles in mice engrafted with human AML cells shows a significantly longer survival compared to control nanoparticles or free miR-29b (106).

**Figure 1:**
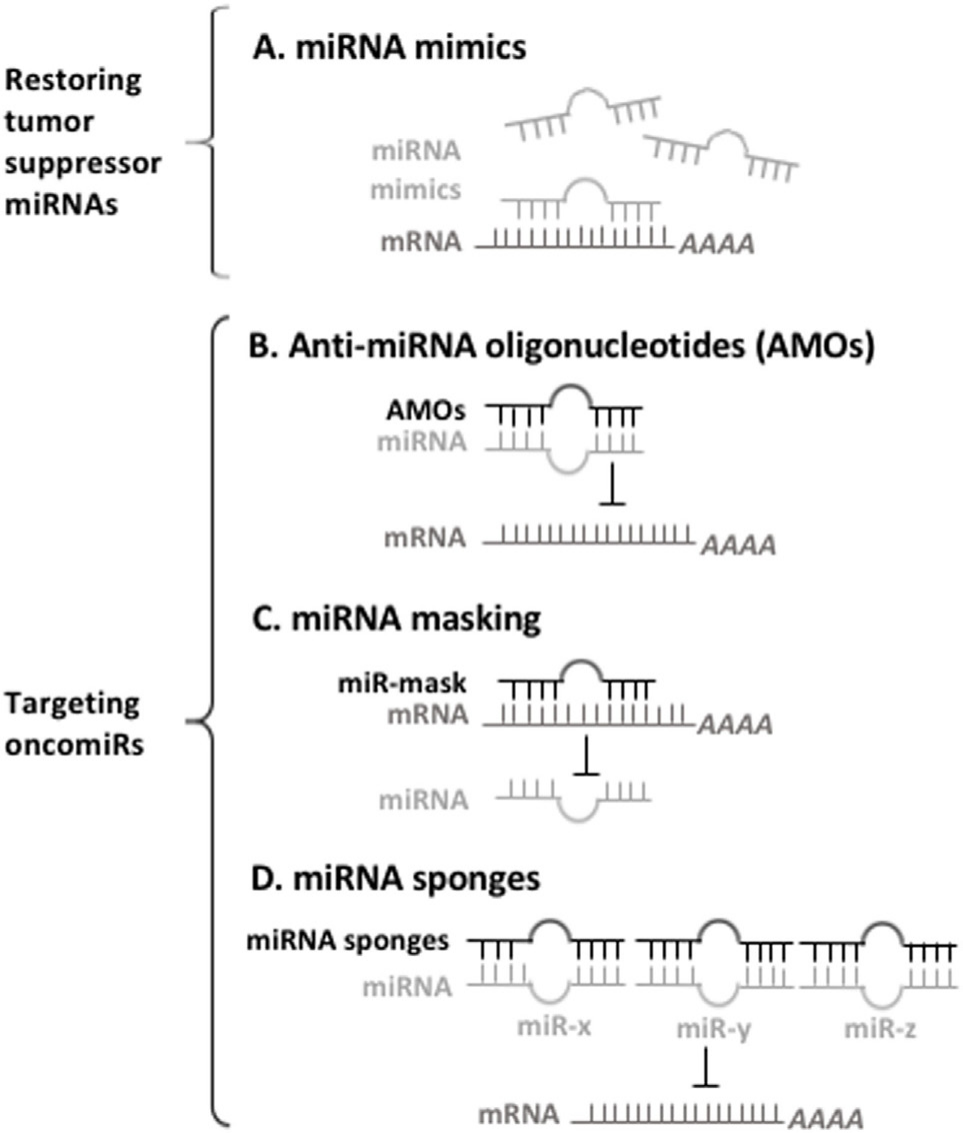
A prospect of miRNA-based therapy for myeloid malignancies. **(A)** Synthetic oligonucleotides used for restoring the depleted microRNAs (miRNAs) bind to their target mRNAs for inhibiting mRNAs of oncogenes. **(B)** Anti-miRNA oligonucleotides (AMOs) interact with oncomiRs, thus preventing them from interacting with their target mRNA. **(C)** miRNA-mask is designed to bind to 3′UTR of mRNAs, thus preventing oncomiRs recognition their target mRNAs. **(D)** miRNA sponges have multiple complementary sites against targeted miRNA, thereby inhibit the functions of oncomiRs.

Conversely, targeting overexpressed oncomiRs can be conducted mainly by three approaches: anti-miRNA oligonucleotides (AMOs; antagomiRs), miRNA masking, and miRNA sponges (Figure 1). AMOs that are chemically modified with the locked nucleic acid (LNA) can be systemically delivered to affect cancer-related pathway *via* the binding and inhibiting oncomiRs in leukemia. For instance, targeting nanoparticles containing miR-126 antagonists (antagomiR-126) results in an *in vivo* reduction of leukemic stem cells by depletion of the quiescent cell sub-population (74). miR-21- and miR-196b-specific antagomiRs inhibit *in vitro* leukemic colony-forming activity and *in vivo* leukemia-initiating cell activity of HOX-based leukemias, which have led to improved survival and delayed disease onset in murine AML models (107). miRNA-masking antisense oligonucleotides (miR-mask) can be used to achieve a gene-specific anti-miRNA therapy that masks the specific target mRNA from endogenous miRNA, and thus prevent the inhibitory action of miRNA. miRNA sponges are another approach to silence miRNAs with potentially important clinical utility, and have complementary binding sites to seed sequences of target miRNAs. This advantage gives them the ability to inhibit multiple miRNAs that have the same sequence in their seed region. It has been shown that using miR-22 sponges, both the leukemic cell proliferation and the activity of miR-22 are markedly impaired (60).

In addition to these encouraging outcomes with the use of miRNA-based therapy in preclinical, animal models, the first therapies targeting miRNAs have now entered clinical trials (108). Treatment of an LNA inhibitor of miR-122 (known as Miravirsen) in patients with hepatitis C virus infection holds great promise of miRNA-based therapeutics (https://ClinicalTrials.gov, NCT01200420) (109). Furthermore, the first cancer-targeted miRNA drug—MRX34, a liposome-based miR-34 mimic—has entered clinical trials in patients with advanced or metastatic liver cancer (https://ClinicalTrials.gov, NCT01829971) (110). These studies provide a proof of principle that should encourage future endeavors of miRNA-directed therapy for leukemia.

## Conclusion and Perspectives

There are many more miRNAs, shown in publication, that are involved in the pathogenesis of myeloid malignancies, suggesting intense enthusiasm for research in this area in recent years. However, the regulatory changes in miRNA levels are often small and might get lost in the biological noise when using a small number of samples. Using *in vitro* systems to study the miRNA phenotypes might be different from what happens *in vivo*. Also, the efficacy of overexpression or antagomiR tools should be validated using downstream target readout to convince the endogenous interaction between the miRNA and the targets.

MicroRNAs have emerged as the potential targets for therapeutic applications. Circulating miRNAs in exosomes/extracellular vesicles from serum or plasma represent a new source of promising biomarkers that may be applied to clinical settings. A specific MDS/AML-associated serum miRNA profiles could not only provide an exciting screening tool for early detection of leukemia in the clinic but could also be used to track leukemic blasts relapsed after chemotherapy. However, exosomal miRNAs loaded from leukemic cells can be transferred to stromal or normal HSC recipient cells and alter their functions, thereby promoting leukemic phenotypes. A further investigation of the relevance of exosomal miRNAs to the pathogenesis of myeloid malignancies is clearly warranted.

Although the case of miRNA-based therapeutics entering clinical trials continues to grow, no miRNA-based therapy has yet made its way to clinical trials particularly for the treatment of AML. A main obstacle of applying miRNA-based therapeutics for clinical use is the limitation of more efficient and specific delivery methods. Thus, many new approaches are currently being explored for improved delivery of miRNA-based therapies, including liposomes, nanoparticles, LNAs with increased stability, and peptide-based inhibitors. Further, how to precisely deliver miRNA mimics or antagomiRs into the targeted cells *in vivo* has also become another major barrier preventing the establishment of miRNA-directed strategies. But nevertheless, miRNA-based therapies may be available soon for the treatment of myeloid malignancies, and miRNA-based therapeutics may be efficacious when used in a combination with current chemotherapy regimen for leukemia.

## Author Contributions

All authors listed have made a substantial, direct, and intellectual contribution to the work and approved it for publication.

## Conflict of Interest Statement

The authors declare that the research was conducted in the absence of any commercial or financial relationships that could be construed as a potential conflict of interest.
